# SFRP2 enhances the osteogenic differentiation of apical papilla stem cells by antagonizing the canonical WNT pathway

**DOI:** 10.1186/s11658-017-0044-2

**Published:** 2017-08-08

**Authors:** Luyuan Jin, Yu Cao, Guoxia Yu, Jinsong Wang, Xiao Lin, Lihua Ge, Juan Du, Liping Wang, Shu Diao, Xiaomeng Lian, Songlin Wang, Rui Dong, Zhaochen Shan

**Affiliations:** 1 Laboratory of Molecular Signaling and Stem Cells Therapy, Beijing Key Laboratory of Tooth Regeneration and Function Reconstruction, Capital Medical University School of Stomatology, No. 4 Tiantanxili, Dongcheng District, Beijing, 100050 China; 2 Department of Implant Dentistry, Capital Medical University School of Stomatology, Beijing, 100050 China; 3 Molecular Laboratory for Gene Therapy and Tooth Regeneration, Beijing Key Laboratory of Tooth Regeneration and Function Reconstruction, Capital Medical University School of Stomatology, No. 4 Tiantanxili, Dongcheng District, Beijing, 100050 China; 4 Department of Stomatology, Beijing Children’s Hospital, Capital Medical University, No.56 Nanlishi Road, Xicheng District, Beijing, 100045 China; 5 Department of Biochemistry and Molecular Biology, Capital Medical University School of Basic Medical Sciences, No. 10 Xitoutiao Youanmen, Fengtai District, Beijing, 100069 China; 6 Department of Stomatology, Beijing Shijitan Hospital, Capital Medical University, Beijing, 100045 China; 7 Oral and Maxillofacial Surgery Department, Capital Medical University School of Stomatology, No. 4 Tiantanxili, Dongcheng District, Beijing, 100050 China

**Keywords:** SFRP2, Osteogenic differentiation, Stem cells from apical papilla (SCAPs), Wnt signaling, β-catenin

## Abstract

**Background:**

Exploring the molecular mechanisms underlying directed differentiation is helpful in the development of clinical applications of mesenchymal stem cells (MSCs). Our previous study on dental tissue-derived MSCs demonstrated that secreted frizzled-related protein 2 (SFRP2), a Wnt inhibitor, could enhance osteogenic differentiation in stem cells from the apical papilla (SCAPs). However, how SFRP2 promotes osteogenic differentiation of dental tissue-derived MSCs remains unclear. In this study, we used SCAPs to investigate the underlying mechanisms.

**Methods:**

SCAPs were isolated from the apical papilla of immature third molars. Western blot and real-time RT-PCR were applied to detect the expression of β-catenin and Wnt target genes. Alizarin Red staining, quantitative calcium analysis, transwell cultures and in vivo transplantation experiments were used to study the osteogenic differentiation potential of SCAPs.

**Results:**

*SFRP2* inhibited canonical Wnt signaling by enhancing phosphorylation and decreasing the expression of nuclear β-catenin in vitro and in vivo*.* In addition, the target genes of the Wnt signaling pathway, *AXIN2* (axin-related protein 2) and *MMP7* (matrix metalloproteinase-7), were downregulated by *SFRP2*. *WNT1* inhibited the osteogenic differentiation potential of SCAPs. *SFRP2* could rescue this *WNT1*-impaired osteogenic differentiation potential.

**Conclusions:**

The results suggest that SFRP2 could bind to locally present Wnt ligands and alter the balance of intracellular Wnt signaling to antagonize the canonical Wnt pathway in SCAPs. This elucidates the molecular mechanism underlying the SFRP2-mediated directed differentiation of SCAPs and indicates potential target genes for improving dental tissue regeneration.

**Electronic supplementary material:**

The online version of this article (doi:10.1186/s11658-017-0044-2) contains supplementary material, which is available to authorized users.

## Background

Mesenchymal stem cells (MSCs) are considered a good cell source for therapies focused on tissue regeneration [1]. First isolated from the bone marrow, MSCs have since also been successfully obtained from other tissue, like dental tissue, including periodontal ligament stem cells (PDLSCs), dental pulp stem cells (DPSCs), dental follicle stem cells (DFSCs) and stem cells from apical papilla (SCAPs) [1–3]. Displaying the potential to differentiate into various cell types, including odontoblasts, osteoblasts, chondrocytes, myocytes and adipocytes, these cells are capable of self-renewal, are easily accessible and more intimately associated with dental tissues, can generate bone- or dentin-like mineralized tissues, and can repair tooth defects [1–5]. However, their potential clinical applications are limited because the mechanism underlying their directed differentiation remains largely unknown.

Wnts are potent regulatory proteins in stem cells, modulating proliferation and differentiation via both canonical and non-canonical pathways. WNT1, WNT3a and WNT8 can activate the canonical Wnt signaling pathway, which has β-catenin as a key mediator [6]. Some studies have shown that the Wnt/β-catenin pathway enhances osteogenesis of MSCs and osteoprogenitor cells by upregulating osteoblast-related genes [7]. However, the pathway has also been reported to inhibit the osteogenesis capacity of PDLSCs [8]. These findings suggest that the canonical Wnt/β-catenin pathway might play different roles in MSCs derived from different tissues. Thus, delicate control of Wnt signaling is crucial for hemostasis and tissue regeneration.

The family of secreted frizzled-related proteins (SFRPs), which includes five human SFRPs members, binds directly to Wnts to prevent receptor binding and activation of Wnt signaling [9, 10]. It has been shown that SFRP2 could directly modulate proliferation of MSCs. Increased SFRP2 expression in MSCs is associated with enhanced efficacy of MSC therapy in wound granulation and repair of infarcted myocardium [11].

SFRP2 is upregulated during osteogenesis of MSCs [12] and significantly increases ALP activity in C3H10T1/2 cells [13]. In addition, researchers have found that overexpression of SFRP2 increases the survival of MSCs derived from the bone marrow and umbilical cord under oxidative stress [14]. Animal studies also demonstrate that overexpression of SFRP2 could enhance cardiac wound repair during intramyocardial implantation of MSCs [15, 16].

Our previous study demonstrated that SFRP2 could enhance the osteogenic differentiation ability of SCAPs [17], which could enhance the directed differentiation of MSCs for applications in dental tissue regeneration. However, it remains unclear how SFRP2 promotes osteogenic differentiation and whether the canonical Wnt/β-catenin signaling pathway is involved in the process.

In this study, we used SCAPs to investigate the underlying mechanisms for SFRP2 action in osteogenic differentiation. Here, we reveal that SFRP2 inhibits the canonical Wnt/β-catenin signaling by increasing the level of phosphorylated β-catenin, inhibiting the nuclear expression of β-catenin and downregulating the target genes of the Wnt signaling pathway. In addition, SFRP2 could restore the osteogenic differentiation capacity impaired by WNT1 in SCAPs. These findings provide novel insight into the mechanisms underlying SFRP2-mediated directed differentiation of dental tissue-derived MSCs and their potentially valuable clinical applications.

## Methods

### Cell cultures

Dental tissues were obtained under approved guidelines set by the Beijing Stomatological Hospital, Capital Medical University with informed consent from the patients. Wisdom teeth were first disinfected with 75% ethanol and then washed with phosphate buffered saline (PBS). SCAPs were gently separated from the apical papilla of the root and then digested in a solution of 3 mg/ml collagenase type I (Worthington Biochemical Corp.) and 4 mg/ml dispase (Roche Diagnostics Corp.) for 1 h at 37 °C.

Single-cell suspensions were obtained by passing the cells through a 70 μm Falcon strainer (BD Biosciences). MSCs were grown in a humidified, 5% CO2 incubator at 37 °C in DMEM alpha (Invitrogen), supplemented with 15% fetal bovine serum (FBS; Invitrogen), 2 mmol/l glutamine, 100 U/ml penicillin, and 100 μg/ml streptomycin (Invitrogen). The culture medium was changed every 3 days. The phenotype, lineage markers and lineage differentiation potentials of SCAPs were identified in a previous study [18].

Human embryonic kidney 293 T cells were maintained in complete DMEM with 10% FBS (Invitrogen), 100 U/ml penicillin, and 100 μg/ml streptomycin (Invitrogen).

### Plasmid construction and viral infection

The plasmids were constructed according to standard methods. All structures were verified by appropriate restriction digestion and/or sequencing. Human full-length *SFRP2* cDNA was generated using a standard gene synthesis method and sub-cloned into the LV5 lentiviral vector (GenePharma Company). Full-length *WNT1* cDNA fused to a hemagglutinin (HA) tag was generated using a standard gene synthesis method and sub-cloned into the pLNCX retroviral vector. Short hairpin RNAs (shRNAs) with complementary sequences of target genes were sub-cloned into the pLKO.1 lentiviral vector (Addgene). For viral infections, MSCs were plated overnight and then infected with retroviruses or lentiviruses in the presence of polybrene (6 μg/ml, Sigma-Aldrich) for 6 h. After 48 h, the infected cells were selected with 2 μg/ml puromycin. Scrambled shRNAs (Scramsh) were purchased from Addgene. The target sequence for the shRNAs is: SFRP2 shRNA (SFRP2sh), 5′-ttgatgtaggttatctccttc-3′.

### RT-PCR and real-time PCR

Total RNA was isolated from MSCs with TRIzol reagent (Invitrogen). We synthesized cDNA from 2 μg aliquots of RNA using random hexamers or oligo(dT), and reverse transcriptase according to the manufacturer’s protocol (Invitrogen). Real-time PCR was performed with the QuantiTect SYBR Green PCR kit (Qiagen) and an iCycler iQ Multicolor Real-time PCR Detection System. The primers sequences are:

GAPDH, forward primer, 5′-cgaacctctctgctcctcctgttcg −3′ and reverse primer, 5′-catggtgtctgagcgatgtgg-3′.

AXIN2, forward primer, 5′-ctccccaccttgaatgaaga-3′ and reverse primer, 5′-gtttccgtggacctcacact-3′.

MMP7, forward primer, 5′-aaactcccgcgtcatagaaa-3′ and reverse primer, 5′-ttctgcaacatctggcactc-3′.

### Alkaline Phosphatase and alizarin red staining

SCAPs were grown in mineralization-inducing media using the StemPro Osteogenesis Differentiation Kit (Invitrogen). Cells were induced for 5 days and then ALP activity was assayed with an ALP activity kit according to the manufacturer’s protocol (Sigma-Aldrich). Signals were normalized based on protein concentrations.

To detect mineralization, cells were induced to mineralize for 2 weeks, fixed with 70% ethanol, and stained with 2% Alizarin Red (Sigma-Aldrich). To quantitatively determine calcium, Alizarin Red was de-stained with 10% cetylpyridinium chloride in 10 mM sodium phosphate for 30 min at room temperature. The concentration of calcium was determined by measuring the absorbance at 562 nm on a microplate reader and comparing the results to a standard calcium curve generated using calcium dilutions in the same solution. The final calcium level in each group was normalized to the total protein concentration detected in a duplicate plate.

### Nuclear extracts

The nuclear extracts of SCAPs were prepared with a Nuclear Cytosol Extraction Kit (Applygen Technologies Inc.) according to the manufacturer’s instructions. Briefly, 5.0 × 10^6^ SCAPs were harvested by centrifugation. The pelleted cells were resuspended in 250 μl of cytosol extraction buffer A, incubated on ice for 10 min, mixed with 15 μl cytosol extraction buffer B, and incubated on ice for 1 min. The lysates were centrifuged, and the pellets were washed with cytosol extraction buffer A and then resuspended in 50 μl of cold nuclear extraction buffer. After incubation at 4 °C for 30 min with constant rotation, the suspension was spun at 12,000 g at 4 °C for 5 min. The nuclear extract was collected from the supernatant fraction.

### Western blot analysis

Cells were lysed in RIPA buffer consisting of 10 mM Tris–HCl, 1 mM EDTA, 1% sodium dodecyl sulfate (SDS), 1% NP-40, 1:100 proteinase inhibitor cocktail, 50 mM β-glycerophosphate and 50 mM sodium fluoride. The samples were separated on a 10% SDS polyacrylamide gel and transferred to polyvinylidene difluoride (PVDF) membranes with a semi-dry transfer apparatus (Bio-Rad). The membranes were blotted with 5% dehydrated milk for 2 h and then incubated with primary antibodies overnight. The immune complexes were incubated with horseradish peroxidase-conjugated anti-rabbit or anti-mouse IgG (Promega) and visualized with SuperSignal reagents (Pierce). Primary antibodies against HA (clone no. C29F4, cat. no. 3724, Cell Signaling Technology), phosphorylated β-catenin (p-β-catenin, cat. no. ab38511, Abcam), and β-catenin (β-catenin, clone no. D10A8, cat. no. 8480 s, Cell Signaling Technology). We also used a primary monoclonal antibody to detect the housekeeping protein beta-actin (β-actin, cat no. C1313, Applygen Company) or histone H3 (cat. no. sc10809, Santa Cruz Biotechnology).

### Transplantation in nude mice

This study was approved by the Animal Care and Use Committee of Beijing Stomatological Hospital, Capital Medical University. The animal experiments carried out in accordance with the National Institutes of Health guidelines for the care and use of laboratory animals (NIH Publications No. 8023, revised 1978). Animals were purchased from the Institute of Animal Science of the Vital River Co., Ltd. No drugs or previous procedures were used. Approximately 4.0 × 10^6^ cells were mixed with 40 mg of HA/tricalcium phosphate ceramic particles (Engineering Research Center for Biomaterials), and then transplanted beneath the dorsal skin of 10-week old immunocompromised beige mice (nu/nu nude mice). These procedures were performed according to the approved animal protocol. Eight weeks later, the implants were harvested, fixed with 10% formalin, decalcified with 10% EDTA buffer (pH 8.0), and embedded in paraffin. To detect the expression of phosphorylated β-catenin, sections were stained immunohistochemically using antibody against phosphorylated β-catenin (p-β-catenin, cat. no. ab38511, Abcam).

### Transwell cultures

Six Transwell plates were used in this study. Chambers with a 0.4 μm pore size membranes (Corning) were used to physically separate the SCAP-SFRP2 from the SCAP-HA-WNT1 cells. Approximately 2 × 10^5^ SCAP-SFRP2 cells or SCAPs transfected with empty vector (SCAP-vector1) were seeded in the upper chamber, and 2 × 10^5^ SCAP-HA-WNT1 cells or SCAPs transfected with empty vector (SCAP-vector2) were placed in the bottom chamber in the presence of DMEM alpha supplemented with 15% fetal bovine serum, 2 mmol/l glutamine, 100 U/ml penicillin and 100 μg/ml streptomycin. After 24 h, the culture medium was changed to mineralization-inducing medium using the StemPro Osteogenesis Differentiation Kit.

### Statistics

All statistical calculations were performed with SPSS 13. Student’s t test was performed to determine statistical significance. *p* ≤ 0.05 was considered significant.

## Results

### SFRP2 inhibited the canonical Wnt signaling in SCAPs

**Fig. 1:**
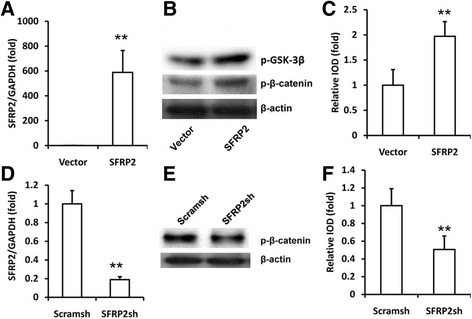
SFRP2 increased the expression of phosphorylated β-catenin in SCAPs. **a** Real-time RT-PCR results confirmed the overexpression of *SFRP2* via lentivirus infection in SCAPs. *GAPDH* was used as an internal control. **b** Western blot results showed that the overexpression of *SFRP2* increased the expression of phosphorylated β-catenin and phosphorylated GSK-3β. Beta-actin was used as an internal control. **c** Quantitative analysis of phosphorylated β-catenin based on western blot results. **d** Real-time RT-PCR detection of *SFRP2* expression after SCAPs were infected with short hairpin RNAs (shRNA) that silenced *SFRP2* (*SFRP2sh*) compared with scrambled shRNA (Scramsh). *GAPDH* was used as an internal control. **e** Western blot results showed that depletion of *SFRP2* downregulated the expression of phosphorylated β-catenin. **f** Quantitative analysis of phosphorylated β-catenin based on western blot results. Beta-catenin was used as an internal control. Student’s t test was performed to determine statistical significance. All error bars represent SD (*n* = 3). **p ≤ 0.01

*SFRP2-*overexpressing constructs were transduced into SCAPs via lentiviral infection. Ectopic *SFRP2* expression was confirmed using real-time RT-PCR (Fig. 1a). We detected the phosphorylation of β-catenin in SCAPs. The results showed that overexpression of *SFRP2* increased the expression of p-β-catenin and p-GSK-3β in SCAPs (Fig. 1b and c). However, overexpression of *SFRP2* did not affect the expression of *WNT5a*, which is a representative of the non-canonical wnt signaling pathway (Additional file 1: Fig. S1). During the process of osteogenesis, *SFRP2* increased the expression of p-β-catenin compared to the level seen in control SCAPs (Additional file 2: Fig. S2).

Then, we designed a short hairpin RNA (shRNA) to target *SFRP2* and introduced it into SCAPs via lentiviral infection (SCAP-*SFRP2sh* cells). After selection, knockdown efficiency (90%) was verified using real-time RT-PCR (Fig. 1d). We discovered that the silencing of *SFRP2* downregulated the expression of phosphorylated β-catenin in SCAPs (Fig. 1e and f).

**Fig. 2:**
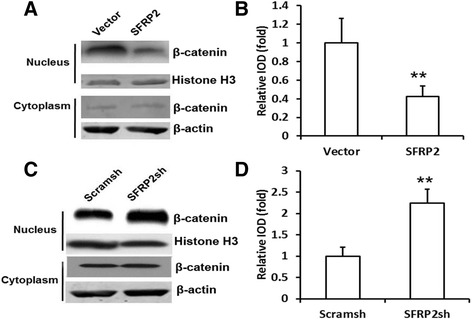
SFRP2 inhibited β-catenin into the nuclei of SCAPs. **a** Western blot results showed that overexpression of *SFRP2* decreased the level of nuclear β-catenin. **b** Quantitative analysis of nuclear β-catenin based on western blot results. **c** Western blot results showed that the depletion of *SFRP2* upregulated the level of nuclear β-catenin. Beta-actin or histone H3 was used as an internal control. **d** Quantitative analysis of nuclear β-catenin based on western blot results. Student’s t test was performed to determine statistical significance. All error bars represent SD (*n* = 3). **p ≤ 0.01

Next, nuclear β-catenin level was detected using western blot analysis. The results showed that overexpression of *SFRP2* decreased the expression of nuclear β-catenin in SCAPs (Fig. 2a and b). Silencing *SFRP2* upregulated the expression of nuclear β-catenin in SCAPs (Fig. 2c and d).

**Fig. 3:**
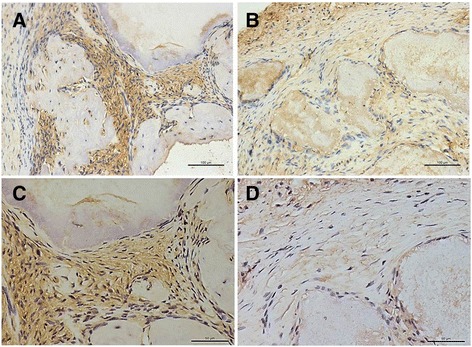
SFRP2 enhanced the phosphorylation of β-catenin in vivo. Immunohistochemical analysis showed that the overexpression of SCAP-SFRP2 increased the phosphorylation of β-catenin (**a**, **c**), SCAP-vector was used as the control (**b**, **d**). Scale bars = 100 μM (**a**, **b**) and 50 μM (**c**, **d**)

We then subcutaneously transplanted the SCAP-*Vector* or SCAP-*SFRP2* cells into nude mice. The transplanted tissues were harvested 8 weeks after transplantation. Immunohistochemistry staining results revealed more phosphorylated β-catenin in the tissues transplanted with SCAP*-SFRP2* cells (Fig. 3a and c) than in the tissues transplanted with SCAP-*Vector* cells (Fig. 3b and d). These results show that overexpression of SFRP2 significantly enhanced the phosphorylation of β-catenin.

**Fig. 4:**
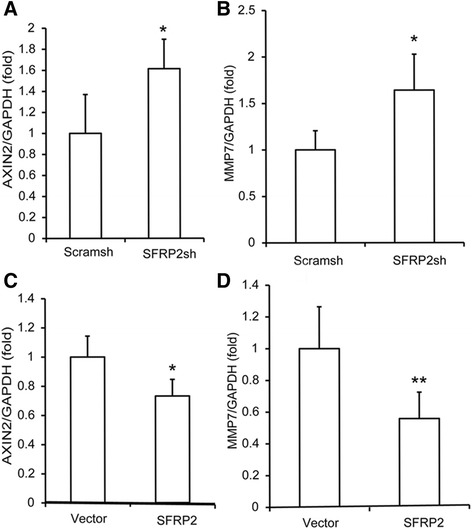
SFRP2 decreased the expression of Wnt target genes in SCAPs. **a**, **b** Real-time RT-PCR showed that the depletion of *SFRP2* upregulated the expression of *AXIN2* and *MMP7*. **c**, **d** Real-time RT-PCR results showed that the overexpression of *SFRP2* decreased the expression of *AXIN2* and *MMP7.* Student’s t test was performed to determine statistical significance. All error bars represent SD (*n* = 3). *p ≤ 0.05, **p ≤ 0.01

After overexpression or knockdown of *SFRP2* (without osteogenesis induction), the levels of Wnt target genes, including *C-myc*, *AXIN2*, *DKK1* and *MMP7*, were detected using real-time PCR. The results showed that silencing *SFRP2* enhanced the expression of *AXIN2* and *MMP7* (Fig. 4a and b), while overexpression of SFRP2 downregulated the expression of *AXIN2* and *MMP7* (Fig. 4c and d). However, the expression of *C-myc* and *DKK1* did not change regardless of overexpression or knockdown of *SFRP2* in SCAPs (data not shown).

### WNT1 inhibited the osteogenic differentiation potential of SCAPs

**Fig. 5:**
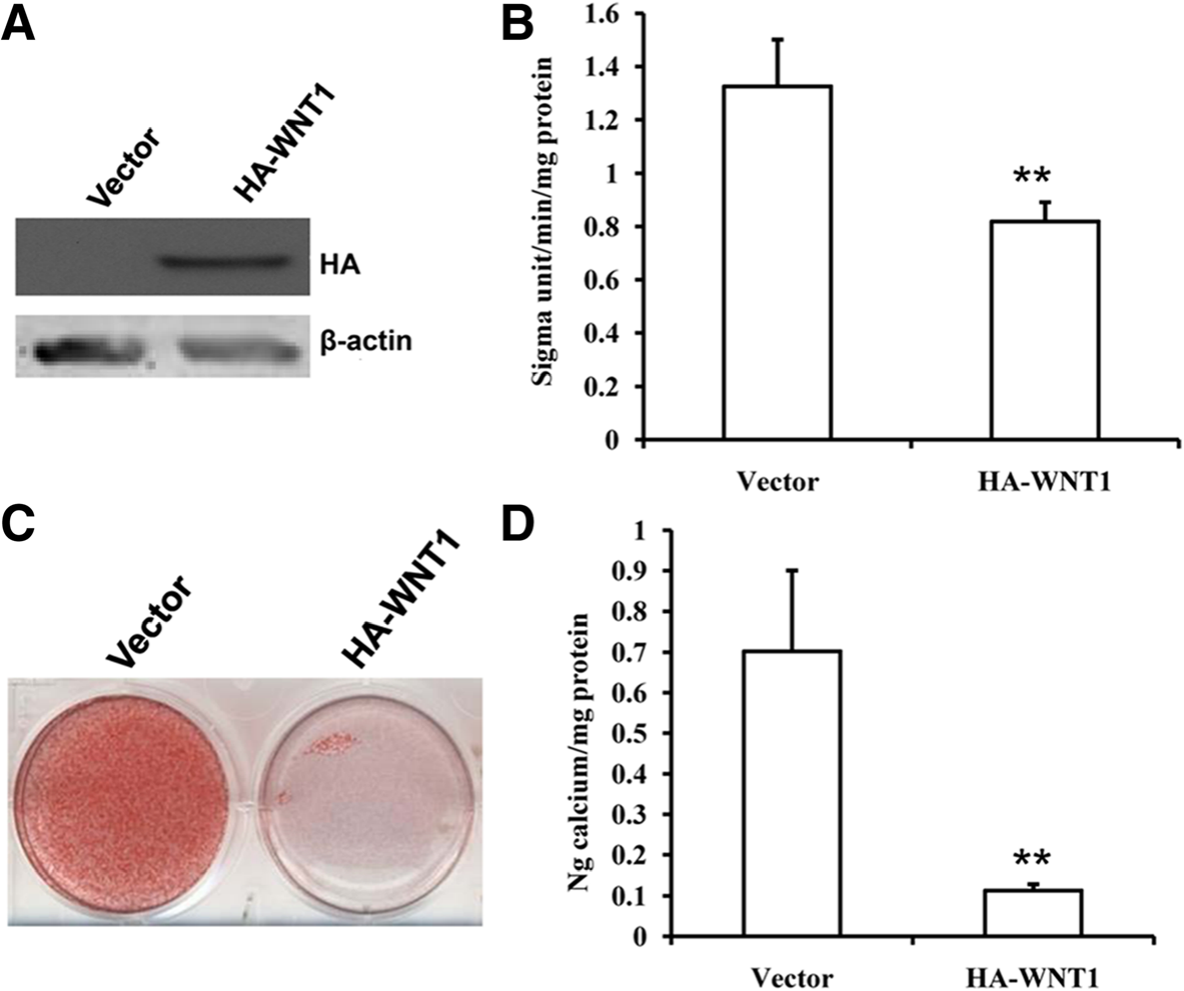
Overexpression of *WNT1* inhibited the osteogenic differentiation capacity of SCAPs. **a** Western blot results confirmed the overexpression of *WNT1* via retrovirus infection of SCAPs. Beta-actin was used as an internal control. **b** ALP activity. **c** Alizarin Red staining. **d** Quantitative analysis of calcium concentration. Student’s t test was performed to determine statistical significance. All error bars represent SD (*n* = 3). **p ≤ 0.01. (sigma unit: unit of measurement for ALP activity)

We inserted the *WNT1*-expressing sequence into a retroviral vector. This construct overexpressing ectopic *WNT1* was transduced into SCAPs via retroviral infection. Ectopic *WNT1* expression was confirmed using western blot analysis (Fig. 5a). After induction for 5 days, we found that overexpression of wild-type *WNT1* decreased ALP activity in SCAPs (Fig. 5b). Two weeks after induction, mineralization was markedly inhibited in SCAPs with overexpressed *WNT1* compared with that in cells infected with the empty vector, as determined by Alizarin Red staining and quantitative calcium measurements (Fig. 5c and d).

### SFRP2 rescued the WNT1-impaired osteogenic differentiation potential in SCAPs

**Fig. 6:**
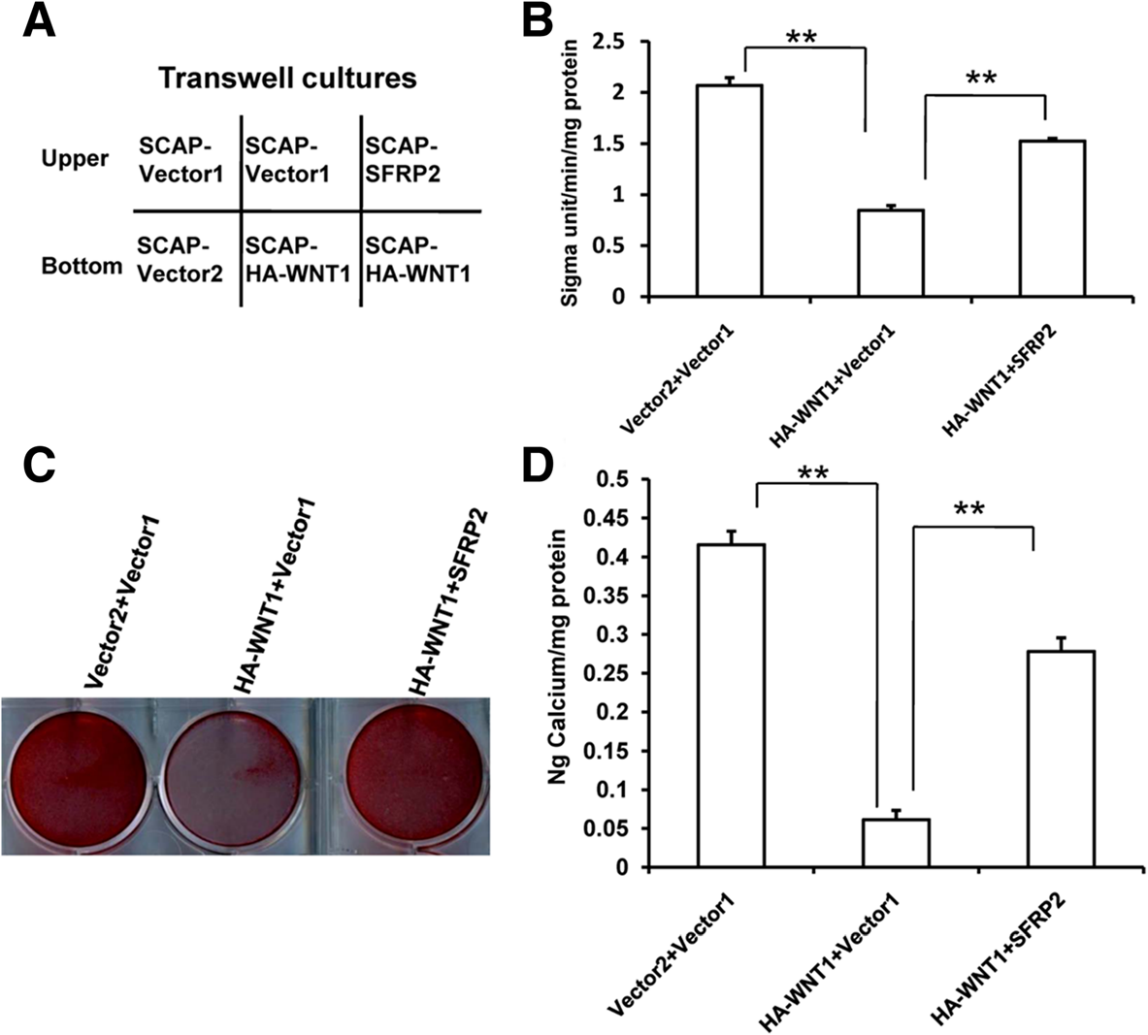
SFRP2 rescued the impaired osteogenic differentiation potential of WNT1 in SCAPs. **a** Schematic representation of the transwell culture. **b** Transwell culture results showed that overexpression of *SFRP2* could partially inhibit the reduction in ALP activity by WNT1. **c** Alizarin Red staining and (**d**) quantitative analysis of calcium concentration results showed that the overexpression of *SFRP2* could partially inhibit the reduction in mineralization by WNT1. Vector 1: empty vector as the control of SFRP2. Vector2: empty vector as the control of HA-WNT1. Student’s t test was performed to determine statistical significance. All error bars represent SD (*n* = 3). **p ≤ 0.01. (sigma unit: unit of measurement for ALP activity)

A transwell experiment was used to investigate the effect of *SFRP2* on the *WNT1*-mediated osteogenic differentiation potentials of SCAPs (Fig. 6a). Interestingly, we found that overexpression of *SFRP2* could partially inhibit the decrease in ALP activity induced by *WNT1* (Fig. 6b). Alizarin Red staining and quantitative calcium measurements consistently revealed that the *WNT1*-weakened mineralization markedly recovered in SCAPs that overexpressed *SFRP2* compared with the recovery in cells infected with the empty vector (Fig. 6c and d). Besides, ALP activity and quantitative analysis of calcium concentration results also showed that 100 ng/ml SFRP2 recombinant protein could enhance the osteogenic capacity of SCAPs (Additional file 3: Fig. S3).

## Discussion

MSCs derived from dental tissues have been demonstrated to be a promising source of material for cell therapy. However, little is known about the molecular mechanism that maintains the stability and differentiation potential of MSCs. It is known that the commitment and differentiation of MSCs into osteocytes, chondrocytes and adipocytes requires Wnt/β-catenin signaling [19–22].

It has been reported that the loss of function of LRP5 (low-density lipoprotein receptor-related protein), which is a co-receptor for Wnt, leads to a reduction in bone mass. However, a gain-of-function mutation in LRP5 causes an autosomal dominant disorder characterized by high bone density [23, 24]. Another study has shown that inhibiting the expression of β-catenin could efficiently diminish the expression of the early osteogenic marker ALP induced by Wnt3a in SCAPs [25]. These results strongly suggest that canonical Wnt/β-catenin signaling may play an important role in osteogenic signaling in MSCs.

SFRPs play a pivotal role in the Wnt pathway and mainly function as antagonists of Wnt signaling [26, 27]. Previous research demonstrated that SFRP2 could physically bind to Wnt3a and inhibit the transcriptional activities of β-catenin/TCF within hypoxic cardiomyocytes in a dose-dependent manner and could enhance the survival response of cardiomyocytes against hypoxia-induced apoptosis [28]. Boland et al. showed that exogenous WNT3a could inhibit the osteogenic differentiation of MSC based on increased β-catenin nuclear localization and activation of a Wnt-responsive promoter, but also that this effect could be partially attenuated by SFRP3 [12]. SFRP4 has also been reported to gradually increase during the late stages of adipogenic differentiation in hAMSCs and has been shown to correlate with the inhibition of canonical Wnt signaling, as demonstrated by the inhibition of β-catenin expression [29]. Elevated β-catenin protein levels were detected at low SFRP1 concentrations, which were reduced at high concentrations of SFRP1 in C57MG cells [30].

In this study, we found that *SFRP2* could enhance the phosphorylation of β-catenin and GSK-3β, and that it could inhibit the expression of nuclear β-catenin at the protein level. Accordingly, *SFRP2* also affected the expression of Wnt target genes including *AXIN2* and *MMP7*. Upregulation of *SFRP2* could inhibit the expression of *AXIN2* and *MMP7* and vice versa. These results suggest that SFRP2 could act as an antagonist of the canonical Wnt signaling pathway in SCAPs. However, under certain circumstances, SFRP2 has been shown to display a synergistic effect on Wnt/β-catenin signaling, functioning as an active agonist of Wnt16b, facilitating cancer cell proliferation, migration and drug resistance [31]. In other studies, SFRP2 has been shown to either increase or decrease β-catenin stabilization in different cellular contexts (stimulation of Wnt3a activity in HEK293 and C57MG cells but inhibition in L cells) [32, 33].

Besides, this has shown that SFRP2 had no effect on wnt5a expression at the mRNA level. It is speculated that SFRP2 might not affect the non-canonical wnt pathway. For other cell types and disease models, SFRP2 may exert different effects. SFRP2 has been shown to inhibit the proliferation of cardiac progenitor cells and prime them for cardiac differentiation by modulation of both canonical and non-canonical Wnt/Planar Cell Polarity (PCP) pathways through JNK [34]. Thus, we cannot exclude that cell-specific actions are at play, which might explain the complexity of the effects of SFRP2 on the Wnt pathway.

Our previous study demonstrated that *SFRP2* was upregulated upon osteogenic differentiation of MSCs derived from dental tissue and bone marrow in a time-dependent manner. Moreover, gain-of-function studies showed that *SFRP2* enhanced osteo/dentinogenic differentiation of SCAPs and the expression of the *BSP*, *COL1A2*, *OPN*, *DSPP* and *DMP1* genes, which encode different extracellular matrix proteins of the bone and dentin. Moreover, *SFRP2* was able to activate the expression of *OSX* independent of the transcription factors *RUNX2*. In addition, transplantation experiments demonstrated that *SFRP2* considerably enhanced osteo/dentinogenesis in vivo [17].

We also found that low dose of SFRP2 recombinant protein (100 ng/ml) could effectively enhance the osteogenesis capacity of SCAPS, which may provide a simple and convenient means for clinical use. Consistently, this study showed that more phosphorylated β-catenin was detected in SCAP-*SFRP2* cells during the process of osteogenesis and SCAP-*SFRP2* cell transplanted samples, indicating that the Wnt signaling pathway was inhibited during osteogenesis process [17, 35].

Subsequently, we assessed whether the effect of SFRP2 in promoting osteogenesis was mediated by the Wnt pathway. Indeed, overexpression of *WNT1* decreased the osteogenic differentiation capacity of SCAPs. Furthermore, *SFRP2* could rescue the *WNT1*-mediated impaired osteogenic differentiation in SCAPs in a competitive manner.

However, SFRP2 may also display an inverse effect on bone formation. SFRP2 protein has been reported to be widely expressed by multiple myeloma (MM) cells from patients. In MM, cell-derived SFRP2 plays an important role in the suppression of bone formation in advanced stages of the disease [36]. In addition, SFRP2 was found to be strongly expressed in ameloblastoma tissues and AM-1 cells. After the depletion of SFRP2, the AM-1 cells showed diffuse mineralization [37]. Furthermore, upregulated expression of *SFRP2* has been implicated in the suppression of osteoblast activity during inflammation-induced bone loss, and it was shown to vary with the progression of inflammation [38, 39]. In addition, SFRP2 was shown to protect mouse MSCs from apoptosis under hypoxic conditions through inhibition of canonical Wnt signaling without affecting the osteogenic differentiation potential of MSCs [14, 16]. These reports showed that the effects of SFRP2 on bone regeneration are different in pathophysiological states, indicating comprehensive roles of SFRP2.

## Conclusions

Our results showed that *SFRP2* enhanced the osteogenic differentiation in SCAPs by inhibiting the canonical Wnt signaling pathway. Our work explored the molecular mechanisms underlying directed differentiation of MSCs mediated by *SFRP2* and provided potential target genes for improving tissue regeneration mediated by dental tissue-derived MSCs.

## Additional files

Additional file 1: Figure S1. Overexpression of *SFRP2* did not affect the expression of *WNT5a* in SCAPs. (TIFF 1872 kb)

Additional file 2: Figure S2. *SFRP2* increased the expression of p-β-catenin during the process of osteogenesis. A – Expression of phosphorylated β-catenin increased in control SCAPs on days 7 and 10 during the osteogenic process. Overexpression of SFRP2 increased the expression of phosphorylated β-catenin. B – Quantitative analysis of p-β-catenin at different times based on western blot results. Student’s t test was performed to determine statistical significance. All error bars represent SD (*n* = 3). *p ≤ 0.05. (TIFF 1833 kb)

Additional file 3: Figure S3. SFRP2 recombinant protein enhanced the osteogenic capacity of SCAPs. A – ALP activity and B – quantitative analysis of calcium concentration results showed that 100 ng/ml SFRP2 recombinant protein could enhance the osteogenic capacity of SCAPs. All error bars represent SD (*n* = 3). *p ≤ 0.05. (sigma unit: unit of measurement of ALP activity). (TIFF 1174 kb)

## Abbreviations

AXIN2: axin-related protein 2
DFSCs: Dental follicles stem cells
DPSCs: Dental pulp stem cells
GAPDH: Glyceraldehyde 3-phosphate dehydrogenase
MMP7: Matrix metalloproteinase-7
MSCs: Mesenchymal stem cells
PDLSCs: Periodontal ligament stem cells
SCAPs: Stem cells from apical papilla
SFRP2: Secreted frizzled-related protein 2
