# Supplementary figures and images for: SFRP2 enhances the osteogenic differentiation of apical papilla stem cells by antagonizing the canonical WNT pathway
Source: Cell Mol Biol Lett. 2017 Aug 8;22:14. doi: 10.1186/s11658-017-0044-2 (PMC5547503; doi:10.1186/s11658-017-0044-2)

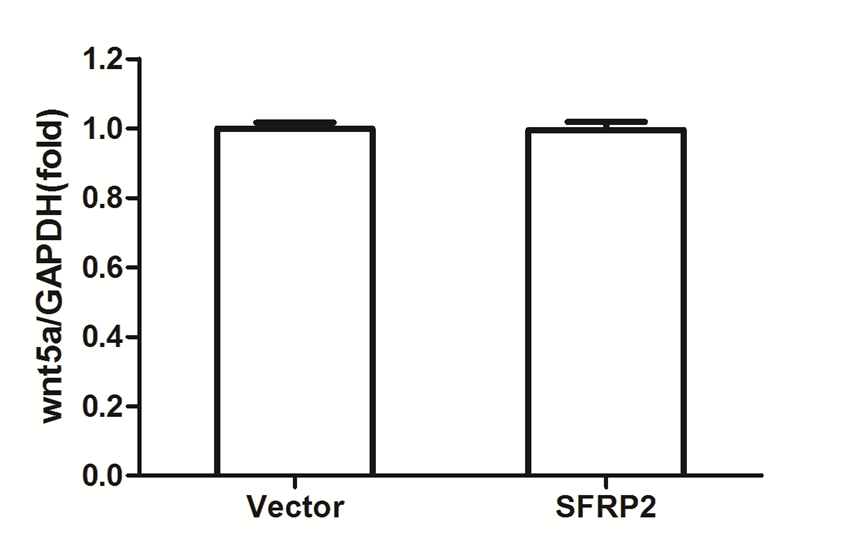

Supplement: Supplementary file 1 — Overexpression of SFRP2 did not affect the expression of WNT5a in SCAPs. (TIFF 1872 kb) [file 11658_2017_44_MOESM1_ESM.tif]

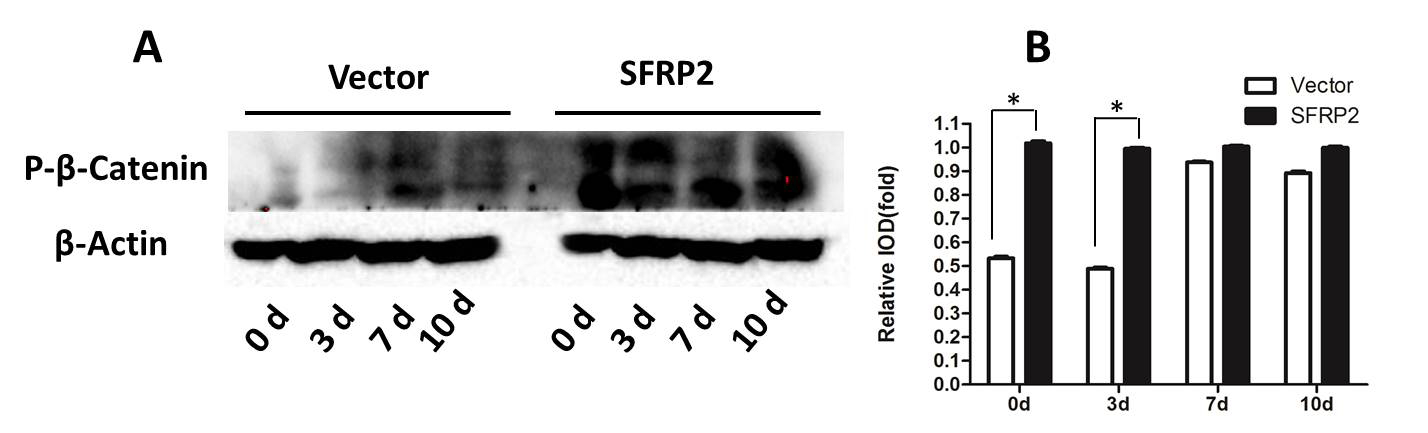

Supplement: Supplementary file 2 — SFRP2 increased the expression of p-β-catenin during the process of osteogenesis. A – Expression of phosphorylated β-catenin increased in control SCAPs on days 7 and 10 during the osteogenic process. Overexpression of SFRP2 increased the expression of phosphorylated β-catenin. B – Quantitative analysis of p-β-catenin at different times based on western blot results. Student’s t test was performed to determine statistical significance. All error bars represent SD (n = 3). *p ≤ 0.05. (TIFF 1833 kb) [file 11658_2017_44_MOESM2_ESM.tif]

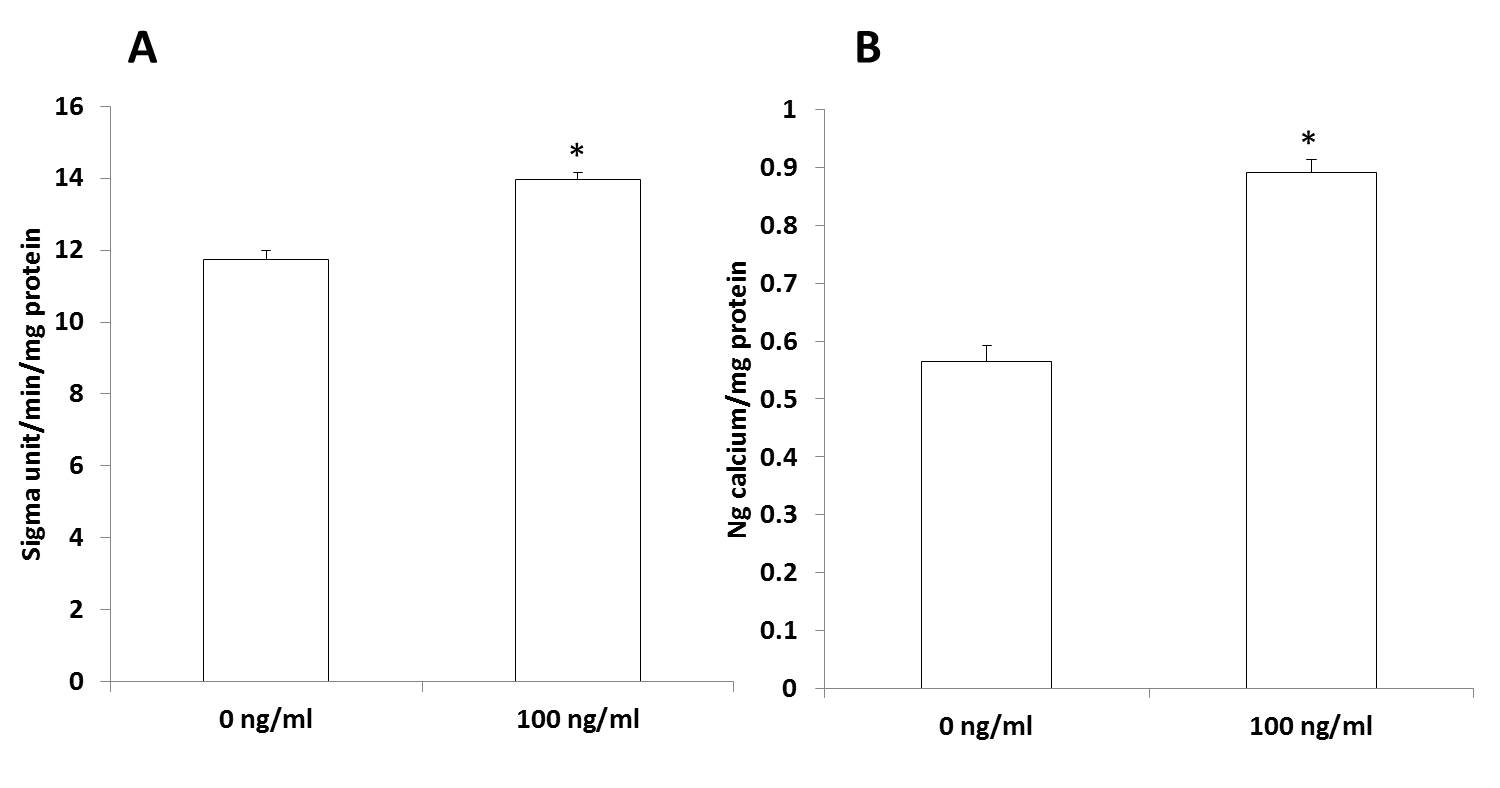

Supplement: Supplementary file 3 — SFRP2 recombinant protein enhanced the osteogenic capacity of SCAPs. A – ALP activity and B – quantitative analysis of calcium concentration results showed that 100 ng/ml SFRP2 recombinant protein could enhance the osteogenic capacity of SCAPs. All error bars represent SD (n = 3). *p ≤ 0.05. (sigma unit: unit of measurement of ALP activity). (TIFF 1174 kb) [file 11658_2017_44_MOESM3_ESM.tif]
